# The victimisation experience schedule: contextualising interpersonal trauma and perceived discrimination in individuals with psychotic experiences with and without a need-for-care

**DOI:** 10.1007/s00127-025-02917-0

**Published:** 2025-04-30

**Authors:** I. Verdaasdonk, M. A. Charalambides, D. Baumeister, M. Jackson, P. A. Garety, C. Morgan, T. Ward, E. Peters

**Affiliations:** 1 Department of Psychosis Research and Innovation, Parnassia Psychiatric Institute, The Hague, The Netherlands; 2 Department of Clinical, Neuro & Developmental Psychology, Faculty of Behavioral and Movements Sciences, Vrije Universiteit, Amsterdam, The Netherlands; 3 Institute of Psychiatry, Department of Psychology, Psychology and Neuroscience, King’s College London, London, UK; 4 Department of General Internal Medicine and Psychosomatics, University Hospital Heidelberg, Heidelberg, Germany; 5 School of Psychology, Bangor University, Bangor, North Wales UK; 6 Betsi Cadwaladr University Health Board, Bangor, North Wales UK; 7 NIHR Biomedical Research Centre for Mental Health, South London and Maudsley NHS Foundation Trust, London, UK; 8 Institute of Psychiatry, Psychology and Neuroscience, Health Service & Population Research, King’s College London, London, UK; 9 South London & Maudsley NHS Foundation Trust, London, UK

**Keywords:** Victimisation, Psychotic experiences, Need-for-care, Contextual factors

## Abstract

**Purpose:**

Victimisation is associated with psychotic experiences (PEs) across the psychosis continuum, yet contextual factors possibly influencing outcomes have been neglected. Building on the Unusual Experiences Enquiry study (UNIQUE) showing higher childhood trauma but lower discrimination in individuals with PEs without a need-for-care, compared to those with a need-for-care, this study utilized a novel instrument to examine victimisation-related contextual factors.

**Methods:**

Individuals from the UNIQUE study with persistent PEs with (clinical, *n* = 82) and without (non-clinical, *n* = 92) a need-for-care, and a control group without PEs (*n* = 83), completed the Victimisation Experiences Schedule (VES). This multidimensional instrument, comprising items from validated measures, assesses interpersonal traumas and discriminatory experiences, alongside contextual factors: impact, powerlessness, social support, age, duration, frequency, victim-perpetrator relationships, and reasons for discrimination.

**Results:**

There were no differences in lifetime interpersonal traumas between the clinical and non-clinical groups, with the latter reporting slightly more than controls. The clinical group experienced more lifetime perceived discrimination than the other groups. No differences emerged in impact and powerlessness at the time of victimisation; however, the clinical group reported lower positive social support and higher current impact and powerlessness for both types of victimisation. Discrimination occurred earlier and lasted longer in the clinical group than the other groups, often attributed to mental health and race/ethnicity, likely reflecting a higher proportion of racially minoritized individuals.

**Conclusion:**

The results suggest an interplay between risk and protective factors around victimisation that may shape outcomes, highlighting the importance of assessing contextual factors of victimisation using comprehensive tools like the VES.

**Supplementary Information:**

The online version contains supplementary material available at 10.1007/s00127-025-02917-0.

## Introduction

Psychotic experiences (PEs) are common in the general population (prevalence rates 6–15%) without always necessitating psychiatric care [1, 2], supporting the continuum view of psychosis [3]. Approximately 20% of people with PEs report persistent experiences in the absence of distress, prior psychotic disorder diagnosis, or help-seeking from mental health services in relation to their PEs, i.e., they do not have a “need-for-care” [1]. Although individuals with (i.e. clinical) and without (i.e. non-clinical) a need-for-care show similarities in PE-phenomenology, those with distress exhibit more cognitive difficulties, paranoia, negative symptoms, and threat-based appraisals [4–7]. These findings align with cognitive models of psychosis, suggesting that the appraisal of PEs as uncontrollable or being caused by others is pivotal in the transition to clinical psychosis [8, 9].

Adverse life events, particularly victimisation experiences, are hypothesised to influence the appraisal of PEs by shaping negative schemata about oneself and others [8–10]. Growing evidence indicates an association between victimization and PEs [11, 12], with some studies suggesting a causal relationship [13, 14]. Specifically, interpersonal traumas during childhood and adolescence, like bullying, parental neglect, and sexual, physical, or psychological abuse [15–17], as well as perceived discrimination in adulthood [18–20] have been linked with PEs. These associations are found in both clinical and non-clinical groups [17, 21–23]. Victimisation experiences do not predict need-for-care status [24, 25], with similar rates of trauma exposure reported in both groups [5, 6, 26–28], suggesting that victimisation is associated with the occurrence of PEs rather than clinical outcome.

A key challenge in the literature is the lack of variables that differentiate clinical and non-clinical groups reliably in terms of victimisation, underlining the need for exploration of contextual or psychological factors that may determine benign or pathological outcomes. Previous studies suggest that impact and powerlessness persist in individuals with need-for-care, potentially due to less use of adaptive coping strategies in response to victimisation experiences compared to individuals with need-for-care [26, 29, 30]. Moreover, experiences of social defeat (e.g., bullying or social exclusion) and subsequent feelings of powerlessness in interpersonal relationships can be mirrored in the phenomenology of PEs, which in turn is linked to distress [31, 32]. In contrast, positive social support following trauma can buffer against detrimental psychological outcomes [33, 34]. When such support is lacking, or when negative social responses such as unwanted advice or unsympathetic behaviour are present, the impact of trauma exposure may be exacerbated through maladaptive cognitive appraisals, coping, and avoidance [35]. In general, individuals with need-for-care report receiving less social support than those without [23, 24, 36]. However, it remains unclear whether social support specifically around victimisation experiences differ between individuals with and without need-for-care.

Other factors related to victimisation may also be relevant to the risk of developing PE-related need-for-care. For example, early age and high frequency of exposure have been linked to more detrimental outcomes than later and less exposure [37]. Specifically, Offen and colleagues found an association between malevolence of voices and sexual abuse at an early age [38]. Additionally, the nature of the victim-perpetrator relationship may play a role in developing a psychotic disorder. However, current evidence is limited and mixed, with both abuse by mothers [39], and by people other than parents [36] having been found to lead to an increased risk of psychosis.

Despite previous findings implicating the role of contextual factors in the association between victimisation and need-for-care, previous research has been limited by methodology, typically using comparatively crude measures that focus on incidence and neglect context. To address this gap, the Victimisation Experiences Schedule (VES) was developed by drawing items from various validated measures to form a comprehensive instrument, capturing a wide range of interpersonal traumas and discriminatory experiences, with the addition of relevant contextual variables. A recent study [40] demonstrated excellent inter-rater reliability and adequate convergent validity of the German version of the VES, highlighting its utility in assessing trauma across a spectrum of severity. Moreover, a subset of data from the VES in the Unusual Experiences Enquiry (UNIQUE) study showed that the non-clinical group reported marginally more childhood interpersonal trauma than controls but not the clinical group, while the clinical group reported significantly more lifetime discrimination than both groups [6].

The current study employed the full VES instrument to test group differences in contextual factors related to victimisation experiences, particularly focusing on impact, powerlessness, and social support, as well as age, duration, frequency, victim-perpetrator relationships, and reasons for discrimination.

## Methods

### Design

The current study is part of the UNIQUE study [6], a cross-sectional design comparing clinical, sociodemographic, and psychological characteristics in individuals across the psychosis continuum from urban (South London) and rural (Bangor, North Wales) areas in the UK. It was approved by the London-Westminster National Research Ethics Service Committee (12/LO/0766); the South London and Maudsley NHS Foundation Trust/King’s College London Institute of Psychiatry, Psychology and Neuroscience Research and Development (R&D2012/047); and the Betsi Cadwaladr University Health Board Research and Development (Jackson/LO/0766).

Previous papers [6, 41] published a subset of VES data. This paper presents, for the first time, comprehensive contextual data from the VES.

### Participants

A power analysis was conducted in relation to the main outcomes of the UNIQUE study, but not the current, exploratory study.

Three groups were recruited: (1) individuals with need-for-care diagnosed with a psychotic disorder (i.e. clinical); (2) individuals with persistent PEs without a need-for-care (i.e., non-clinical); (3) controls without PEs or need-for-care, broadly matched to non-clinical group in age, gender, ethnicity, and education. Exclusion criteria for all groups included: (a) aged < 18 years; (b) insufficient English language proficiency; (c) primary substance dependence; (d) history of neurological disease, brain injury, or epilepsy.

### Inclusion criteria

The clinical group was recruited from inpatient and outpatient services of the South London and Maudsley NHS Foundation Trust and Betsi Cadwaladr University Health Board. Inclusion criteria were a psychotic disorder diagnosis (ICD-10 categories F20-39 [42]) and current positive symptoms (scoring ≥2 on at least one item of the Scale for Assessment of Positive Symptoms [SAPS] [43]).

The non-clinical group consisted of self-selected individuals recruited through specialist sources, including advertisements on spiritualist and psychic forums, a central research register, and a circular advert distributed on the King’s College London email list. Inclusion criteria were: (a) presenting current positive symptoms (scoring ≥2 on at least one item of the SAPS [43]); (b) reporting one or more PEs on the Psychosis Screening Questionnaire (PSQ) [44], and ‘occasional’ (i.e., at least monthly) positive or first-rank Schneiderian symptom experiences on the Unusual Experiences Screening Questionnaire (UESQ) [45] (c) presenting PEs for more than 5 years to exclude individuals in prodromal stages; (d) never contacted mental health services/GPs for their PEs (nor anyone else on their behalf); (e) never contacted secondary mental health services; and (f) no PE-related distress as indicated by a score of < 2 (i.e., ‘unmet need’) on basic self-care and psychological distress items of the Camberwell Assessment of Need Short Appraisal Schedule (CANSAS) [46], and as judged by research workers.

Controls were either volunteered by participants in the non-clinical group or recruited through research registers or community advertisements. Inclusion criteria were: (a) no PEs (i.e., endorsed no items on UESQ or PSQ); (b) scoring ±1 standard deviation from the Unusual Experiences subscale mean of the Oxford-Liverpool Inventory of Feelings and Experiences (O-LIFE) [47]; and (c) never contacted secondary mental health services.

## Measures

### Screening measures

The Psychosis Screening Questionnaire (PSQ) [44] and Unusual Experiences Screening Questionnaire (UESQ) [45] were used to screen participants in the non-clinical and control groups for PEs. The PSQ assesses PEs in the preceding year across five domains (hallucinations, hypomania, paranoia, strange experiences, and thought disorder), indicating experiences may be symptoms of psychosis. The screening tool UESQ, derived from the AANEX [45], comprises nine items establishing the presence of positive and first-rank Schneiderian symptoms in the last month in clear consciousness and in the absence of drug use.

The Unusual Experiences subscale of the Oxford-Liverpool Inventory of Feelings and Experiences (O-LIFE) [47] was used to screen the control group for phenomenologically related positive symptoms of psychosis (see above section ‘Participants’). This subscale comprises 30 items on perceptual aberrations, magical thinking, and hallucinations, with responses recorded as “yes” or “no”, resulting in a total score ranging from 0 to 30.

The Camberwell Assessment of Need Short Appraisal Schedule (CANSAS) [46] was used to screen for clinical and social needs in the non-clinical group. Only items 1–4 (regarding accommodation, food, home, and self-care) and item 9 (psychological distress) were administered, scoring items from 0 to 2 (0 = no problem; 1 = met need; 2 = unmet need).

### Sociodemographic and clinical measures

IQ was estimated using the Wechsler Adult Intelligence Scale - short form, 3rd edition (WAIS-III) [48].

The clinical and non-clinical groups completed the Appraisals of Anomalous Experiences (AANEX) [45] semi-structured interview to assess current PEs and associated cognitive and emotional correlates. Only scores on the first part of the interview (AANEX-Inventory, short form [28]) are reported, measuring presence of 17 anomalous experiences across the lifetime (‘have you ever experienced?’) and currently (‘in the past month’) on a 3-point scale (1 = not present; 2 = unclear; 3 = present). Age of onset and duration of PEs were also reported.

The SAPS [43] and the SANS [49] were used to assess severity and frequency of positive and negative symptoms. The SAPS comprises 35 items, and the SANS 25 items, all rated 0–5 (‘None’-‘Severe’), with total scores ranging from 0 to 175 and 0-125, respectively.

### Victimisation experiences schedule (VES)

The VES is a semi-structured interview covering two categories of victimisation experiences: (a) interpersonal trauma, (b) perceived discrimination. It comprises 14 items extracted from validated measures (Childhood Experience of Care and Abuse [CECA] [50]; Trauma History Questionnaire [THQ] [51]; Discrimination Interview [52]; Life Events and Difficulties Schedule [LEDS] [53]). We aimed to capture a comprehensive range of victimisation experiences and contextual variables, without burdening participants (for full measure see Supplementary Material 1). The interview was preceded by an introduction emphasizing confidentiality around disclosing sensitive information, participants’ right not to answer questions, and our intention to approach unusual experiences respectfully without assuming they are caused by victimisation. The final version was informed by feedback on acceptability, feasibility, and relevance gathered at the design stage during consultations with participants with and without a need-for-care with prior experience in similar studies.

#### Category A: interpersonal trauma

This category comprises nine items: five THQ [51] items (sexual abuse; physical abuse; physical attack with or without weapon; bullying). The sexual abuse item was divided into (1) sexual intercourse; (2) unwanted sexual contact; and (3) sexual contact using physical force, with an initial screening query. Three further items were added: threat of assault, psychological abuse, and parental neglect. Items had similar, but slightly less explicit, wording to the THQ. Participants endorsing abuse and/or neglect items were asked to report their relationship with the perpetrator, with response options including mother, father, both parents, sibling, other relative, family friend, peer, authority figure, and other.

#### Category B: perceived discrimination

This category includes five items from the Discrimination Interview [52]: unfairly treated: at work, by the police, by the court system, by neighbours and/or family, and by medical services. Participants were also asked to identify the perceived reason for discrimination: gender, race/ethnicity, sexuality, religion, mental health problems, age, or other.

#### Contextual variables

Participants rated impact (“How much did/does this event/experience affect you at the time/now?”) and powerlessness (“How powerless did/does this event/experience make you feel at the time/now?”) on a 0–10 visual analogue scale (0 = Not at all, 5 = Somewhat, 10 = Totally). The level and nature (positive/negative) of social support at the time of the experience (“Did you tell anyone about it?”) was determined by participants’ verbal report and prompts regarding whether the first person(s) they disclosed to were helpful and/or sympathetic, and how they responded. Both social support dimensions were rated on a 0–3 scale (‘None’-‘High’). See Supplementary Material 1 for the scoring guide.

Three additional contextual variables were rated for endorsed items: age victimisation experiences occurred (childhood: 0–17 years; adulthood: >17 years); duration (number of days); and frequency of exposure (4-point scale ranging from ‘1’=rarely (once or twice) to ‘4’=very frequently (weekly+)).

#### Scoring

Scores are obtained for childhood, adulthood, and lifetime victimisation experiences for both interpersonal trauma and perceived discrimination. Up to three discrete events could be recorded per item for both childhood and adulthood (e.g., a period of bullying between ages 5–6 years and 12–14 years old would constitute two endorsements of that item in childhood). Interpersonal trauma total scores were computed by summing the number of reported events across items 1–9, with total scores ranging from 0 to 27 for childhood and adulthood separately, and 0–54 for lifetime. For perceived discrimination, total scores were obtained by summing the number of reported events across items 11–15, with potential scores ranging from 0 to 15 for childhood and adulthood separately, and 0–30 for lifetime.

For contextual variables, scores were averaged at item level and subsequently at category level to avoid overweighting less severe but more common victimisation events (e.g., bullying vs. sexual abuse).

Only lifetime results are presented here to reduce number of analyses.

### Procedures

Eligibility screening was conducted by telephone or in-person (for inpatients). After providing informed consent, participants completed all measures with MC or trained research workers under her and TW’s supervision, including additional experimental procedures reported elsewhere [6, 7, 54–56]. Participants were debriefed and given a £30 honorarium.

Interviews were audio-recorded with participants’ consent. A subset of recordings representing both study sites and the three groups was selected to assess VES interrater reliability (IRR) (all rated by TW).

### Statistical analysis

The IRR was ascertained by the intra-class correlation coefficient (ICC) for total experiences. Cohen’s (linearly weighted) Kappas (*κ*) were computed to measure raters’ agreement for positive and negative support scores. Results were interpreted following Landis and Koch’s guidelines [57].

Statistical analyses were performed using R (version 4.2.3). Sociodemographic and clinical characteristics were compared across groups using chi-square tests for binary variables, ANOVAs for normally distributed continuous variables, and Mann-Whitney *U* or Kruskal-Wallis tests for non-normally distributed variables.

Kruskal-Wallis tests were used for VES outcomes due to non-normality, followed by post-hoc Dunn tests with Sidak’s correction for three multiple tests to identify individual group differences. Significance level for these exploratory analyses was set at α = 0.05. Effect sizes (ES) are reported as *ε*^*2*^ for overall group differences (0.01 = small, 0.06 = moderate, 0.14 = large; [58]) and *r* for individual group comparisons (0.1 = small, 0.3 = moderate, 0.5 = large; [59]). Multiple response chi-square tests used IBM SPSS (version 28.0.1.0) to examine group differences in victim-perpetrator relationships and reasons for discrimination.

## Results

### Sample characteristics

VES was completed by 257 of the 259 UNIQUE study participants [6]: controls *n* = 83; non-clinical *n* = 92; clinical *n* = 82. Group characteristics are provided in Table 1.

The three groups did not differ on age, migrant status, or current cannabis use. The clinical group had a higher proportion of men, non-white ethnicity, current unemployment, and familial psychosis history than the other two groups. Non-clinical and control groups had more years in education and higher IQ than the clinical group. More non-clinical participants endorsed spirituality and non-traditional religious beliefs than clinical and control participants.

Non-clinical participants reported an earlier age of onset and longer duration of PEs compared to clinical participants, but did not differ on current or lifetime AANEX scores. However, the clinical group reported higher scores than the non-clinical group on the SAPS and SANS.

**Table 1:** Sociodemographic and clinical characteristics of the three groups

Characteristics	Controls (*n* = 83)	Non-clinical (*n* = 92)	Clinical (*n* = 82)	Test statistics
Age, mean (SD)	45.5 (13.3)	46.2 (14.2)	41.8 (12.4)	*H* (2) = 4.84, *p* = 0.09
Gender, n (%)				^*χ2*^ (2) = 31.45, *p* < 0.0001 (clinical > non-clinical = controls)
Male	26 (31.3)	25 (27.2)	54 (65.9)	
Female	57 (68.7)	67 (72.8)	28 (34.1)	
Site, n (%)				*χ*^*2*^ (2) = 0.54, *p* = 0.76
Bangor	40 (48.2)	41 (44.6)	41 (50)	
London	43 (51.8)	51 (55.4)	41 (50)	
Ethnicity, n (%)				White vs. others: *χ*^*2*^(2) = 17.61, *p* < 0.001 (clinical < non-clinical = controls)
White	75 (90.4)	80 (87.0)	55 (67.1)	
Mixed	2 (2.4)	3 (3.2)	4 (4.9)	
Asian	2 (2.4)	2 (2.2)	2 (2.4)	
Black	3 (3.6)	6 (6.5)	20 (24.4)	
Other	1 (1.2)	1 (1.1)	1 (1.2)	
Migrant, n (%)	10 (12.1)	14 (15.2)	21 (25.6)	^*χ2*^ (2) = 5.77, *p* = 0.06
Education (years), mean (SD)	17.1 (4.0)*	16.8 (4.2)	14.8 (5.8)	*H* (2) = 26.21, *p* < 0.0001 (clinical < non-clinical = controls)
Currently employed, n (%)	65 (78.3)	64 (69.6)	14 (17.1)	^*χ2*^ (2) = 73.93, *p* < 0.0001 (clinical < non-clinical = controls)
Family history of psychosis, n (%)	4 (4.8)†	9 (9.8)‡	18 (22.0)§	*χ*^*2*^ (2) = 14.73, *p* = 0.001 (clinical > non-clinical = controls)
Religion, n (%)				^*χ2*^ (4) = 68.29, *p* < 0.001 (clinical ≠ non-clinical ≠ controls)
None	48 (57.8)	32 (34.8)	15 (18.3)	
Mainstream	28 (33.7)	19 (20.7)	54 (65.9)	
Non-traditional	7 (8.4)	41 (44.6)	13 (15.8)	
Spiritual, n (%)	34 (41.0)	82 (91.1)¶	61 (74.4)†	*χ*^*2*^ (2) = 54.62, *p* < 0.001 (non-clinical > clinical > controls)
Cannabis use, n (%)				
Past	34 (41.0)	31 (33.7)	44 (53.7)	*χ*^*2*^ (2) = 7.18, *p* = 0.03 (clinical > non-clinical; clinical = controls; non-clinical = controls)
Current	4 (4.8)	2 (2.2)	8 (9.8)	*χ*^*2*^ (2) = 4.93, *p* = 0.08
IQ, mean (SD)	112.2 (16.5)*	105.4 (14.0)¶	85.1 (14.2)‡	*F*_*2,247*=_71.07, *p* < 0.001 (clinical < non-clinical < controls)
Age of onset PEs, mean (SD)		15.1 (12.3)	21.6 (10.4)	*U* = 6495, *p* < 0.001
Length of time with PEs, mean (SD)		31.2 (15.3)	20.2 (12.7)	*U* = 9578.5, *p* < 0.001
AANEX, mean (SD)				
Total current		28.6 (5.1)	30.1 (6.2)¶	*U* = 7471.5, *p* = 0.14
Total lifetime		34.8 (4.9)	36.3 (6.4)*	*U* = 7465.5, *p* = 0.1
SAPS (Total), mean (SD)		12.3 (7.2)*	27.2 (15.4)	*U* = 5618.5, *p* < 0.001
SANS (Total), mean (SD)		3.0 (3.3)†	22.7 (14.1)**	*U* = 4254.5, *p* < 0.001

Note. * One participant missing. † Three participants missing. ‡ Four participants missing. § 11 participants missing. ¶ Two participants missing. ** Six participants missing

*Abbreviations*: AANEX, Appraisals of Anomalous Experiences Interview; PE, psychotic experience; SAPS, Scale for Assessment of Positive Symptoms; SANS, Scale for Assessment of Negative Symptoms

### VES interrater reliability

IRR was completed on 13.6% of the sample (*n* = 35; clinical = 12, non-clinical = 11, controls = 12) for variables prone to interviewer bias, i.e., determining number of valid victimisation experiences and social support ratings. Absolute agreement for total number of reported experiences was excellent (ICC *r* = 0.99). Agreement across social support ratings was substantial for positive support (Agreement = 87.6%; κ = 0.71, standard error (SE) = 0.06) and moderate for negative support (Agreement = 88.9%; κ = 0.51, SE = 0.05).

### Prevalence of victimisation experiences

Overall, 91.8% of our sample reported at least one lifetime interpersonal trauma (controls 90.4%; non-clinical 94.6%; clinical 90.2%), with 85.2% reporting at least one event in childhood (controls 84.3%; non-clinical 89.1%; clinical 81.7%), and 62.6% in adulthood (controls 56.6%; non-clinical 69.6%; clinical 61.0%).

For perceived discrimination, 60.3% of our sample reported at least one lifetime experience (controls 50.6%; non-clinical 58.7%; clinical 72.0%), with 10.5% experiencing at least one event during childhood (controls 7.2%; non-clinical 10.9%; clinical 13.4%) and 58.4% during adulthood (controls 49.4%; non-clinical 55.4%; clinical 70.7%). Further results on group differences in number of victimisation experiences in childhood and adulthood are presented in Table S1, Supplementary Material 1.

### Number of victimisation experiences across lifetime

There was an overall group difference in number of lifetime interpersonal trauma events (*p* = 0.04, *ε*^*2*^ = 0.025), with the non-clinical group (M = 4.75, SD = 3.83) reporting higher scores than the control group (M = 3.52, SD = 2.23; small ES). No differences were found between clinical (M = 3.87, SD = 3.18) and control or non-clinical groups. Group differences for number of lifetime discrimination experiences were also found (*p* < 0.001, *ε*^*2*^ = 0.063), with the clinical group (M = 1.87, SD = 1.68) reporting more discriminatory events than the non-clinical group (M = 1.18, SD = 1.37; small ES) and controls (M = 0.96, SD = 1.23; small-moderate ES). No difference was found between the non-clinical and control group.

### Contextual factors of victimisation experiences

#### Impact and powerlessness at the time and currently (Table 2)

No group differences were found for impact and powerlessness at the time of victimisation for either interpersonal trauma or perceived discrimination.

There were, however, group differences for current impact (*p* < 0.005, *ε*^*2*^ = 0.050) and powerlessness (*p* < 0.001, *ε*^*2*^ = 0.087) of interpersonal trauma. The same pattern was found for perceived discrimination (impact: *p* < 0.001, *ε*^*2*^ = 0.058; powerlessness: *p* = 0.03, *ε*^*2*^ = 0.028). The clinical group reported higher scores than the non-clinical and control groups for both types of events (small-moderate ES), with no differences between the latter two groups.

**Table 2:** Impact and powerlessness of victimisation experiences across lifetime

	Controls (*n* = 83) *	Non-clinical (*n* = 92) †	Clinical (*n* = 82) ‡				Test statistics
**Impact – at the time**							
Interpersonal trauma: mean (SD); median (IQR)	7.13 (1.90); 7.67 (2.28)	7.36 (1.92); 8.00 (2.54)		7.30 (2.13); 7.83 (2.94)		*H* (2) = 1.05, *p* = 0.59, *ε*^*2*^ = 0.004 Control vs. non-clinical: *z*=-0.97, *p*_*adj*_*=*0.701, *r* = 0.063; Control vs. clinical: *z*=-0.78, *p*_*adj*_*=*0.820, *r* = 0.051; Non-clinical vs. clinical: *z* = 0.17, *p*_*adj*_*=*0.998, *r* = 0.011	
Perceived discrimination: mean (SD); median (IQR)	7.24 (2.07); 8.00 (3.00)	7.64 (1.99); 8.00 (2.46)		7.17 (2.51); 7.60 (4.50)		*H* (2) = 1.07, *p* = 0.59, ε^*2*^ = 0.004 Control vs. non-clinical: *z*=-0.98, *p*_*adj*_*=*0.695, *r* = 0.079; Control vs. clinical: *z*=-0.29, *p*_*adj*_*=*0.988, *r* = 0.023; Non-clinical vs. clinical: *z* = 0.76, *p*_*adj*_*=*0.832, *r* = 0.061	
**Impact - currently**							
Interpersonal trauma: mean (SD); median (IQR)	2.04 (2.05); 2.00 (2.25)	2.27 (2.07); 1.69 (2.92)		3.53 (2.76); 3.25 (3.54)		*H* (2) = 12.85, *p* < 0.005, *ε*^*2*^ = 0.050 Control vs. non-clinical: *z*=-0.70, *p*_*adj*_*=*0.861, *r* = 0.046; Control vs. clinical: *z*=-3.36, *p*_*adj*_*=*0.002, *r* = 0.218; Non-clinical vs. clinical: *z*=-2.94, *p*_*adj*_*=*0.009, *r* = 0.191	
Perceived discrimination: mean (SD); median (IQR)	2.18 (2.20); 2.00 (4.00)	2.42 (2.56); 2.00 (4.00)		4.12 (2.89); 4.83 (4.00)		*H* (2) = 14.74, *p* < 0.001, *ε*^*2*^ = 0.058 Control vs. non-clinical: *z*=-0.39, *p*_*adj*_*=*0.973, *r* = 0.031; Control vs. clinical: *z*=-3.36, *p*_*adj*_*=*0.002, *r* = 0.270; Non-clinical vs. clinical: *z*=-3.18, *p*_*adj*_*=*0.004, *r* = 0.256	
**Powerlessness – at the time**							
Interpersonal trauma: mean (SD); median (IQR)	6.97 (2.41); 7.67 (3.42)	7.16 (2.25); 7.56 (2.76)		6.50 (2.71); 7.00 (3.92)		*H* (2) = 2.16, *p* = 0.34, *ε*^*2*^ = 0.008 Control vs. non-clinical: *z*=-0.34, *p*_*adj*_*=*0.982, *r* = 0.022; Control vs. clinical: *z* = 1.05, *p*_*adj*_*=*0.648, *r* = 0.068; Non-clinical vs. clinical: *z* = 1.43, *p*_*adj*_*=*0.395, *r* = 0.093	
Perceived discrimination: mean (SD); median (IQR)	6.95 (2.51); 8.00 (2.83)	7.06 (2.82); 8.00 (4.50)		6.99 (3.11); 8.00 (4.63)		*H* (2) = 0.41, *p* = 0.81, *ε*^*2*^ = 0.002 Control vs. non-clinical: *z*=-0.42, *p*_*adj*_*=*0.965, *r* = 0.034; Control vs. clinical: *z*=-0.64, *p*_*adj*_*=*0.893, *r* = 0.051; Non-clinical vs. clinical: *z*=-0.22, *p*_*adj*_*=*0.995, *r* = 0.018	
**Powerlessness - currently**							
Interpersonal trauma: mean (SD); median (IQR)	1.16 (1.44); 0.50 (2.06)	1.05 (1.46); 0.44 (1.65)		2.87 (2.79); 2.00 (4.63)		*H* (2) = 22.20, *p* < 0.001, *ε*^*2*^ = 0.087 Control vs. non-clinical: *z* = 0.29, *p*_*adj*_*=*0.988, *r* = 0.019; Control vs. clinical: *z*=-3.87, *p*_*adj*_*=*0.0003, *r* = 0.252; Non-clinical vs. clinical: *z*=-4.30, *p*_*adj*_*=*0.00005, *r* = 0.279	
Perceived discrimination: mean (SD); median (IQR)	1.62 (2.03); 0.62 (3.13)	1.72 (2.52); 0.92 (2.66)		2.82 (2.65); 2.50 (5.00)		*H* (2) = 7.18, *p* = 0.03, *ε*^*2*^ = 0.028 Control vs. non-clinical: *z* = 0.01, *p*_*adj*_*=*0.999, *r* = 0.001; Control vs. clinical: *z*=-2.19, *p*_*adj*_*=*0.083, *r* = 0.177; Non-clinical vs. clinical: *z*=-2.36, *p*_*adj*_*=*0.053, *r* = 0.190	
**Social support - negative**							
Interpersonal trauma: mean (SD); median (IQR)	0.32 (0.56); 0.00 (0.42)	0.24 (0.39); 0.00 (0.39)		0.31 (0.52); 0.00 (0.50)		*H* (2) = 0.25, *p* = 0.88, *ε*^*2*^ = 0.001 Control vs. non-clinical: *z* = 0.39, *p*_*adj*_*=*0.972, *r* = 0.025; Control vs. clinical: *z*=-0.07, *p*_*adj*_*=*0.999, *r* = 0.004; Non-clinical vs. clinical: *z*=-0.46, *p*_*adj*_*=*0.956, *r* = 0.030	
Perceived discrimination: mean (SD); median (IQR)	0.50 (0.73); 0.00 (1.00)	0.38 (0.78); 0.00 (0.50)			0.40 (0.58); 0.00 (0.50)	*H* (2) = 1.69, *p* = 0.43, *ε*^*2*^ = 0.007 Control vs. non-clinical: *z* = 0.99, *p*_*adj*_*=*0.690, *r* = 0.080; Control vs. clinical: *z*=-0.13, *p*_*adj*_*=*0.999, *r* = 0.010; Non-clinical vs. clinical: *z*=-1.21, *p*_*adj*_*=*0.534, *r* = 0.098	
**Social support - positive**							
Interpersonal trauma: mean (SD); median (IQR)	0.99 (0.83); 0.88 (1.17)	1.28 (0.78); 1.20 (0.96)		0.97 (0.92); 0.75 (1.75)		*H* (2) = 8.77, *p* = 0.01, *ε*^*2*^ = 0.034 Control vs. non-clinical: *z*=-2.35, *p*_*adj*_*=*0.055, *r* = 0.153; Control vs. clinical: *z* = 0.32, *p*_*adj*_*=*0.984, *r* = 0.021; Non-clinical vs. clinical: *z* = 2.68, *p*_*adj*_*=*0.022, *r* = 0.174	
Perceived discrimination: mean (SD); median (IQR)	1.88 (1.03); 2.00 (2.00)	2.11 (0.98); 2.42 (1.19)		1.05 (0.97); 1.00 (1.50)		*H* (2) = 29.22, *p* < 0.001, *ε*^*2*^ = 0.114 Control vs. non-clinical: *z*=-1.06, *p*_*adj*_*=*0.643, *r* = 0.085; Control vs. clinical: *z* = 3.75, *p*_*adj*_*=*0.005, *r* = 0.302; Non-clinical vs. clinical: *z* = 5.17, *p*_*adj*_*=*0.000001, *r* = 0.416	

Note. Sidak-adjusted p-values for three multiple tests are reported for individual group comparisons. Effect sizes for overall group differences are reported as ε^*2*^ (0.01 small, 0.06 moderate, 0.14 large; [58]). Effect sizes for individual group comparisons were calculated as *r=*$\:z/\surd\:n$, where *z*=standardized test statistic, and *n*=number of observations (0.1 small, 0.3 moderate, 0.5 large; [59])

* *n* = 75 for interpersonal trauma and *n* = 42 for perceived discrimination. † *n* = 87 for interpersonal trauma and *n* = 54 for perceived discrimination. ‡ *n* = 76 for interpersonal trauma and *n* = 58 for perceived discrimination

#### Positive and negative social support (Table 2)

Groups differed on positive social support following both interpersonal trauma (*p* = 0.01, *ε*^*2*^ = 0.034) and perceived discrimination (*p* < 0.001, *ε*^*2*^ = 0.114). The non-clinical group received more support than clinical and control groups (small ES) for interpersonal trauma experiences, who did not differ between each other. Clinical participants reported less positive support for perceived discrimination than the non-clinical (moderate-large ES) and control groups (moderate ES), who did not differ from each other.

No group differences were observed for negative support for either interpersonal trauma or perceived discrimination.

#### Age, duration, and frequency of victimisation experiences (Table 3)

There were no group differences for frequency of interpersonal trauma or perceived discrimination. There were, however, group differences in age at time of discriminatory events (*p* < 0.01, *ε*^*2*^ = 0.042). The clinical group experienced discrimination at an earlier age than the control group (small-moderate ES) and the non-clinical group (small ES). No group differences were found for age at time of interpersonal traumatic events.

Similarly, group differences emerged for duration of perceived discrimination (*p* < 0.005, *ε*^*2*^ = 0.050). The clinical group reported longer durations of discrimination than the control (small-moderate ES) and non-clinical (small ES) groups. There were no group differences for duration of interpersonal trauma.

**Table 3:** Age, duration, and frequency of victimisation experiences across lifetime

	Controls (*n* = 83) *	Non-clinical (*n* = 92) †	Clinical (*n* = 82) ‡	Test statistics
**Age (years)**				
Interpersonal trauma: mean (SD); median (IQR)	13.76 (7.82); 11.83 (7.53)	14.87 (7.61); 13.50 (6.25)	14.80 (7.05; 14.00 (7.99)	*H* (2) = 2.41, *p* = 0.30, ε^*2*^ = 0.009 Control vs. non-clinical: *z*=-1.46, *p*_*adj*_*=*0.375, *r* = 0.095; Control vs. clinical: *z*=-1.21, *p*_*adj*_*=*0.536, *r* = 0.078; Non-clinical vs. clinical: *z* = 0.208, *p*_*adj*_*=*0.996, *r* = 0.013
Perceived discrimination: mean (SD); median (IQR)	34.00 (11.58); 31.75 (11.00)	32.16 (13.41); 30.25 (14.69)	26.84 (9.88); 25.00 (11.70)	*H* (2) = 10.84, *p* < 0.01, ε^*2*^ = 0.042 Control vs. non-clinical: *z* = 0.93, *p*_*adj*_*=*0.731, *r* = 0.075; Control vs. clinical: *z* = 3.11, *p*_*adj*_*=*0.006, *r* = 0.251; Non-clinical vs. clinical: *z* = 2.35, *p*_*adj*_*=*0.056, *r* = 0.189
**Duration (days)**				
Interpersonal trauma: mean (SD); median (IQR)	1611.22 (1403.44); 1338.67 (1768.63)	1679.54 (1372.75); 1460.33 (1838.53)	1377.50 (1157.02); 1159.38 (1562.77)	*H* (2) = 1.58, *p* = 0.45, *ε*^*2*^ = 0.006 Control vs. non-clinical: *z*=-0.42, *p*_*adj*_*=*0.965, *r* = 0.027; Control vs. clinical: *z* = 0.79, *p*_*adj*_*=*0.813, *r* = 0.051; Non-clinical vs. clinical: *z* = 1.24, *p*_*adj*_*=*0.514, *r* = 0.081
Perceived discrimination: mean (SD); median (IQR)	437.33 (674.84); 120.00 (555.33)	973.72 (1411.04); 276.50 (1169.00)	1565.66 (1845.11); 1095.42 (2305.18)	*H* (2) = 12.80, *p* < 0.005, ε^*2*^ = 0.050 Control vs. non-clinical: *z*=-1.24, *p*_*adj*_*=*0.514, *r* = 0.101; Control vs. clinical: *z*=-3.45, *p*_*adj*_*=*0.002, *r* = 0.281; Non-clinical vs. clinical: *z*=-2.36, *p*_*adj*_*=*0.053, *r* = 0.192
**Frequency**				
Interpersonal trauma: mean (SD); median (IQR)	2.66 (0.84); 2.67 (1.11)	2.61 (0.77); 2.67 (0.89)	2.74 (0.95); 2.75 (1.47)	*H* (2) = 0.69, *p* = 0.71, *ε*^*2*^ = 0.003 Control vs. non-clinical: *z* = 0.32, *p*_*adj*_*=*0.984, *r* = 0.021; Control vs. clinical: *z*=-0.49, *p*_*adj*_*=*0.948, *r* = 0.032; Non-clinical vs. clinical: *z*=-0.82, *p*_*adj*_*=*0.794, *r* = 0.053
Perceived discrimination: mean (SD); median (IQR)	2.03 (1.09); 1.50 (2.00)	1.97 (1.09); 1.83 (1.50)	2.34 (0.98); 2.33 (1.48)	*H* (2) = 5.26, *p* = 0.07, *ε*^*2*^ = 0.021 Control vs. non-clinical: *z* = 0.38, *p*_*adj*_*=*0.974, *r* = 0.031; Control vs. clinical: *z*=-1.63, *p*_*adj*_*=*0.280, *r* = 0.132; Non-clinical vs. clinical: *z*=-2.17, *p*_*adj*_*=*0.087, *r* = 0.176

Note. Sidak-adjusted p-values for three multiple tests are reported for individual group comparisons. Effect sizes for overall group differences are reported as ε^*2*^ (0.01 small, 0.06 moderate, 0.14 large; [58]). Effect sizes for individual group comparisons were calculated as *r=*$\:z/\surd\:n$, where *z*=standardized test statistic, and *n*=number of observations (0.1 small, 0.3 moderate, 0.5 large; [59])

* *n* = 75 for interpersonal trauma and *n*_*=*_42 for perceived discrimination. † *n* = 87 for interpersonal trauma and *n* = 54 for perceived discrimination. ‡ *n* = 76 for interpersonal trauma and *n* = 58 for perceived discrimination

#### Victim-perpetrator relationships and reasons for discrimination (Table 4)

No group differences were found for victim-perpetrator relationships, but groups differed on reasons for discrimination (*p* < 0.001). Individual chi-square tests revealed that the clinical group were more likely to attribute perceived discrimination to race/ethnicity (20.0%; small-moderate ES) and mental health problems (27.0%; large ES) compared to the non-clinical (12.0% and 4.0% respectively) and control groups (13.8% and 5.2% respectively). The non-clinical group (15.4%; small-moderate ES) reported more discrimination based on gender than the clinical (2.0%) and control groups (5.2%). Importantly, groups differed on these demographics: the clinical group had a higher proportion of men and non-white ethnicity than non-clinical and control groups, who did not differ from each other on these characteristics.

**Table 4:** Victim-Perpetrator relationship and reason for discrimination across lifetime

**Victim-perpetrator relationship,** **n (%)**	**Controls (** ***n*** ** = 83)**		**Non-clinical (** ***n*** ** = 92)**		**Clinical (** ***n*** ** = 82)**		**Statistics ¶**
	Reported by 127 people ^a^	Reported for 203 events ^b^	Reported by 193 people ^a^	Reported for 327 events ^b^	Reported by 133 people ^a^	Reported for 230 events ^b^	^*χ2*^ (18) = 14.87, *p* = 0.67
Both parents	14 (11.0)	16 (7.9)	24 (12.4)	29 (8.9)	11 (8.3)	14 (6.1)	
Mother	27 (21.3)	48 (23.6)	32 (16.6)	68 (20.8)	22 (16.5)	43 (18.7)	
Father	17 (13.4)	27 (13.3)	27 (14.0)	40 (12.2)	21 (15.8)	33 (14.3)	
Sibling	7 (5.5)	9 (4.4)	14 (7.3)	27 (8.3)	10 (7.5)	20 (8.7)	
Other relative	8 (6.3)	15 (7.4)	9 (4.7)	10 (3.1)	8 (6.0)	13 (5.7)	
Family friend	7 (5.5)	8 (3.9)	7 (3.6)	10 (3.1)	8 (6.0)	11 (4.8)	
Peer	9 (7.1)	12 (5.9)	14 (7.3)	25 (7.6)	7 (5.3)	13 (5.7)	
Authority figure	2 (1.6)	2 (1.0)	9 (4.7)	13 (4.0)	3 (2.3)	3 (1.3)	
Other	36 (29.9)	66 (32.5)	57 (29.5)	105 (32.1)	43 (32.3)	80 (34.8)	
**Reason discrimination, n (%)**	Reported by 58 people ^a^	Reported for 79 events ^b^	Reported by 75 people ^a^	Reported for 104 events ^b^	Reported by 100 people ^a^	Reported for 148 events ^b^	^*χ2*^ (14) = 65.41, *p* < 0.001 Gender: χ^*2*^(2) = 8.16, *p* = 0.02, *V* = 0.178 (non-clinical > clinical = controls) Race/ethnicity: χ^*2*^(2) = 9.76, *p* = 0.008, *V* = 0.195 (clinical > non-clinical = controls) Mental health problems: *χ*^*2*^ (2) = 43.42, *p* < 0.001, *V* = 0.411 (clinical > non-clinical = control)
Gender	3 (5.2)	3 (3.8)	11 (14.7)	16 (15.4)	2 (2.0)	2 (1.4)	
Race/ethnicity	8 (13.8)	9 (11.4)	9 (12.0)	11 (10.6)	20 (20.0)	25 (16.9)	
Religion	0 (0)	0 (0.0)	2 (2.7)	2 (1.9)	3 (3.0)	3 (2.0)	
Mental health problems	3 (5.2)	5 (6.3)	3 (4.0)	3 (2.9)	27 (27.0)	41 (27.7)	
Sexuality	3 (5.2)	3 (3.8)	1 (1.3)	1 (1.0)	2 (2.0)	2 (1.4)	
Age	3 (5.2)	3 (3.8)	5 (6.7)	5 (4.8)	8 (8.0)	9 (6.1)	
Other	38 (65.5)	56 (70.9)	44 (58.7)	66 (63.5)	38 (38.0)	66 (44.6)	

Note. Effect sizes for chi-square tests were determined using Cramer’s *V*; 0.07 indicates small effect, 0.21 moderate, and 0.35 large, with two degrees of freedom [59]. ¶ Statistical testing was performed on number of people

^a^ Numbers represent participants reporting specific victim-perpetrator relationships/ reasons for discrimination for at least one event. Since participants could endorse up to three discrete events per item, potentially reporting different victim-perpetrator relationships/ reasons discrimination, total number of people may exceed group size

^b^ Number represents total number of events with reports of victim-perpetrator relationship/reason for discrimination

## Discussion

This study utilized the VES—a novel, comprehensive, and multidimensional instrument based on previously established measures, with strong interrater reliability across most variables—to examine the association between PE-related need-for-care and contextual factors related to victimisation experiences. Although groups did not differ in reported impact or powerlessness at the time of victimisation, the clinical group exhibited greater current impact and powerlessness for both interpersonal trauma and perceived discrimination compared to the non-clinical and control groups, with ES ranging from small to moderate. Additionally, the clinical group reported less positive social support at the time of event for interpersonal trauma and perceived discrimination than the non-clinical and control groups, with ES ranging from small (interpersonal trauma) to moderate-large (perceived discrimination). In contrast, there were no group differences in negative social support. These findings tentatively suggest that the persistence of impact and feelings of powerlessness, along with insufficient positive social support at the time of victimisation, may be associated with PE-related need-for-care.

Our findings are consistent with prior studies demonstrating higher levels of current impact and powerlessness following trauma [26, 29, 30], and less social support in general [23, 24, 36] in individuals with a need-for-care compared to those without. The ongoing impact and powerlessness reported by the clinical group may be mirrored in the phenomenology of PEs, potentially contributing to their distressing nature [60]. Positive social support at the time of victimisation may facilitate emotion regulation and reduce victimisation-related cognitions linked to self-blame, trust, and vulnerability, mitigating the development of maladaptive schemas and threatening appraisals of PEs [8–10], thereby reducing impact and powerlessness over time. The small to moderate ES observed in individual group comparisons for these contextual variables are to be expected given the complex causal pathways involving multiple interacting risk and protective factors implicated in the transition to clinical psychosis [41].

Regarding negative social support, we found no group differences, with all groups scoring notably low. Possibly, participants and/or raters struggled to differentiate between a lack of positive social support (i.e., scoring ‘0’) and actual negative social support with the prompts used (e.g., “Did you tell anyone about it?”, “Were they helpful?”). IRR analysis indicated only moderate agreement for negative social support versus substantial agreement for positive social support, suggesting the latter was better operationalised. For future studies VES prompts could be refined to elicit negative social support (e.g., “Did anyone respond negatively/dismissively upon sharing your experience?”).

Prior studies have reported similar, high trauma exposure rates in clinical and non-clinical groups [26–28], suggesting that victimisation experiences are a general risk factor for PEs. Similarly, our study found no difference in lifetime interpersonal traumas between clinical and non-clinical, with in fact the latter scoring higher than the control group, although ES were small. As previously published [6], the clinical group reported a higher number of lifetime perceived discrimination events (but no higher frequency) compared to the non-clinical and control groups; the present study found that this was driven by higher rates in adulthood within the clinical group (see Table S1 Supplementary materials), who also experienced discrimination at an earlier age and for longer durations. The clinical group reported higher rates of discrimination attributed to ‘race/ethnicity’ and ‘mental health problems’ than the other two groups. While some of the discrimination experienced by the clinical group may be a consequence of need-for-care status rather than a risk factor, the findings are consistent with previous evidence that racial discrimination may be a relevant factor in the route to need-for-care [20, 61, 62]. Yet these results most likely reflect the higher proportion of people from minoritized ethnic groups in our clinical group. Furthermore, the age of PE onset in the clinical group (*M* = 21.6) was prior to that reported for perceived discrimination experiences (*M* = 26.8), suggesting due caution in making any causal inference from discrimination to need-for-care.

Our findings raise implications for research and clinical practice. The VES may serve as a valuable research tool not just for psychosis research but for any population where victimisation experiences and (mental) health outcomes interact [40]. The multidimensional approach of the VES lends itself well to use certain items or sections as standalone measures if a shortened format is desired, which may improve feasibility in specific research contexts. Moreover, the VES enables in-depth analyses on how these experiences affect psychological well-being, offering insights into the development of targeted prevention and intervention strategies. Here, the ongoing impact and powerlessness experienced by the clinical group underscore the importance of trauma-informed care to help process unresolved emotional distress [63], with a focus on fostering supportive social connections, through peer support networks and validating therapeutic alliances [35, 64]. In addition, the powerlessness findings support the importance of psychological interventions focusing on changing the dynamics and relationships between the person and their PEs, such as malevolent voices [65, 66]. Lastly, our findings regarding the incidence of perceived discrimination stress the relevance of clinical supervision and training regarding equity, diversity, and inclusion when treating those with a need-for-care [67].

Several methodological limitations should be considered when interpreting the present, exploratory findings. Longitudinal studies are necessary to clarify the causality between victimisation, PEs, and need-for-care, which cannot be determined by our cross-sectional design. Additionally, the reliance on retrospective self-report introduces potential biases, as current symptomatology (e.g., paranoia), beliefs, and emotional states may affect recall of contextual factors [68] and interpretation of events, particularly regarding perceived discrimination. Integrating use of the VES with the Experience Sampling Method (ESM) [69] could offer valuable insights into impact and powerlessness in daily life and the influence of factors such as low mood or psychological distress [68].

We recruited controls to broadly match the non-clinical group in sociodemographic variables, but there were several demographic differences between the clinical and non-clinical groups, introducing confounding factors. However, characteristics such as gender, IQ, ethnicity are likely inherent of group status and cannot be adequately controlled through analyses of covariance [70]. Future studies utilizing matched case-control designs are warranted, albeit methodologically challenging.

The sample size was not necessarily powered to detect VES differences, since it was determined for the main UNIQUE study. The contextual data were collected from participants who endorsed victimisation events, reflecting variability of exposure within the sample. As a result, statistical power and generalizability of findings across the entire sample are restricted. Lastly, the VES would benefit from further refinement. In addition to revising the negative social support prompt, an item could be added to address day-to-day discriminatory interactions (e.g., those with strangers or involving verbal abuse), providing a more comprehensive assessment of discrimination.

The present findings are preliminary, providing directions for hypothesis-driven research. Future use of the VES could include item-by-item analyses to identify potential differences in contextual factors related to different types of victimisation. For instance, individuals who have experienced physical or sexual assault may feel more powerless and out of control than those who were neglected [26]. Although we found no group differences in victim-perpetrator relationships overall, it is also possible that specific interactions between types of victimisation (e.g., abuse) and perpetrator (e.g., mother) may differentiate groups and warrant further exploration.

In summary, the current study suggests that the VES may be a useful tool for determining contextual factors related to various victimisation experiences. Although all groups reported equally high levels of impact and powerlessness at the time of victimisation experiences, only the clinical group reported a lack of positive social support following victimisation and ongoing impact and powerlessness. Higher rates of perceived discrimination in the clinical group were likely to reflect ethnicity differences across groups, and partly to be a consequence of need-for-care status. These findings highlight the importance of employing instruments like the VES to contextualise victimisation experiences, thereby facilitating a deeper understanding of the complex interplay of risk and protective factors that may determine benign or pathological outcomes.

## Electronic supplementary material

Below is the link to the electronic supplementary material.

Supplementary Material 1

## Data Availability

The data used to support the findings of this study are not openly available due to reasons of sensitivity. Data are however available from the corresponding author upon reasonable request.
