# Supplementary material for: The victimisation experience schedule: contextualising interpersonal trauma and perceived discrimination in individuals with psychotic experiences with and without a need-for-care
Source: Soc Psychiatry Psychiatr Epidemiol. 2025 Apr 30;60(12):2759–72. doi: 10.1007/s00127-025-02917-0 (PMC12594658; doi:10.1007/s00127-025-02917-0)
Supplement: Supplementary file 1 — Supplementary Material 1 [file 127_2025_2917_MOESM1_ESM.docx]

**SUPPLEMENTARY MATERIAL**

**The Victimisation Experience Schedule: Contextualising Interpersonal Trauma and Perceived Discrimination in Individuals With Psychotic Experiences With and Without a Need-for-Care**

***Social Psychiatry and Psychiatric Epidemiology***

**Author information**

**Verdaasdonk, I.^1,2,3*^, Charalambides, M.A.^3*^, Baumeister, D.^3,4^ , Jackson, M.^5,6^, Garety, P.A.^3,7^, Morgan, C.^8^, Ward, T.^3,9^ † & Peters, E.^3,7,9^ †**

^1^Department of Psychosis Research and Innovation, Parnassia Psychiatric Institute, The Hague, the Netherlands;

^2^Department of Clinical, Neuro & Developmental Psychology, Faculty of Behavioral and Movements Sciences, Vrije Universiteit, Amsterdam, The Netherlands; ^3^Institute of Psychiatry, Psychology and Neuroscience, King’s College London, Department of Psychology, London, UK; ^4^Department of General Internal Medicine and Psychosomatics, University Hospital Heidelberg, Heidelberg, Germany; ^5^School of Psychology, Bangor University, Bangor, North Wales, UK; ^6^Betsi Cadwaladr University Health Board, Bangor, North Wales, UK; ^7^NIHR Biomedical Research Centre for Mental Health, South London and Maudsley NHS Foundation Trust, London, UK; ^8^Institute of Psychiatry, Psychology and Neuroscience, King’s College London, Health Service & Population Research, London, UK; ^9^South London & Maudsley NHS Foundation Trust, London, UK

^*^ Joint first author

† Joint last author

**Author for correspondence: Thomas Ward, E-mail:** [**thomas.ward@kcl.ac.uk**](mailto:thomas.ward@kcl.ac.uk)

**VICTIMISATION EXPERIENCES SCHEDULE**

*Introduction to the Task (Note to researchers: The purpose of this introduction is to a) fully inform participants in advance of the sensitive nature of the questions to follow b) be clear about the participants’ right not to answer questions c) reiterate the rationale of asking these questions d) be explicit about confidentiality).*

*OK, we are now going to move on to something different. Hopefully you remember we have discussed that part of the study would involve questions relating to challenging and traumatic events. We are asking these questions to everybody taking part in the study- however we understand that the questions can be quite personal, so it is important to say clearly that you can choose not to answer any questions that make you feel uncomfortable. It is also important to repeat that the information that you give is confidential and your name will be anonymised. The only time we would need to break this confidentiality would be if there was any indication of current risk to yourself or others- in this case we would have a duty of care to disclose this information. If this was to happen we would speak to you about this in the first instance. Have you got any questions about this?*

*Just before we start I want to make it really clear that by asking these questions I am not trying to suggest in any way that people only have mystical/spiritual/ unusual [insert person’s own word] because of past trauma. We know that for some of the people we are talking to, their experiences are not related to traumatic events at all while for others their experiences can actually be very helpful in coping with past difficult events. The idea of the study is to try to understand whether any of these traumatic events make the difference for those people who are distressed by their experiences. We are not assuming anything but we hope that by understanding the role of traumatic events we can find ways to help those who are distressed”.*

**PROMPT QUESTIONS:**

*Note to researchers: following prompts to be used if the participant endorses item*

| **If yes**  **Frequency:** | Can you tell me what happened?  How often did it happen? |  |
| --- | --- | --- |
| **Age:** | How old were you? |  |
| **Duration:** | When did it start? When did it stop? |  |
| **Support:** | **Did you tell anyone about it?** |  |
| **If yes** | When did you first tell someone?  Were they helpful?  Were they sympathetic?  What did they do or say? |  |
| **Impact:** | (Participant rates 0-10 using Response Card) |  |
|  | “How much did this event/experience affect you at the time?”  “How much does this event/experience affect you now?” |  |
| **Powerlessness:** | (Participant rates 0-10 using Response Card) |  |
|  | “Did you feel powerless at the time of this experience?”  “How powerless does this event/experience make you feel now?” |  |
| **Were there any other times that it happened?** | | |
| **If yes** | Repeat above probes |  |

*For all Interpersonal Trauma Items (except Item 1), also ask the following prompt:*

| **Relationship:** | (Participant rates using Response Card)  What was your relationship to the person in this experience? |  |
| --- | --- | --- |

*For all Discrimination Items, also ask the following prompt:*

| **Reason:** | (Participant rates using Response Card) |
| --- | --- |
|  | What do you think the reason was for this? |

**SECTION 1: INTERPERSONAL TRAUMA**

*[note to researcher- only ask prompts if information is not spontaneously given]*

**BULLYING AT SCHOOL/WORK**

| **1.** | 1. I am now going to ask you a few questions about teasing and bullying you may have experienced both in childhood (0-17 years) and adulthood. By the terms teasing and bullying we mean when people of a similar age to you: |  |
| --- | --- | --- |
|  | **Said mean and hurtful things or made fun of you or called you mean and hurtful names; Completely ignored or excluded you from their group of friends or left you out of things on purpose; Hit, kicked or shoved you, or locked you in a room; Told lies or spread rumours about you; Other hurtful things.** (N.B. We don’t call it teasing or bullying when it is done in a friendly or playful way.) |  |

| **Did you have any such experiences? Yes/No** |
| --- |

**If yes, refer to prompts**

**Notes:**

.......................................................................................................................................................................................................................................................................................................................................................................................... ....................................................................................................................................................................................................................................................................................................................................................................................................................................................................................................................................................................................... .................................................................................................................................................................................................................................................................................................................................................................................................................................................................................................................................................................................................................................................................................................................................................................................... .......................................................................................................................................................................................................................................................................................................................................................................................... .............................................................................................................................................................................................

| **Childhood (0-17 years)** | **Age** | **Frequency** | **Duration**  **(Years/Months)** | **Impact**  **(0-10)**  **Then Now** | | **Support**  **(+)**  **(0-3)** | **Support**  **(-)**  **(0-3)** | **Powerlessness**  **(0-10)**  **Then Now** | | **Anomalous Experience**  **(Pre/Post/ Both)** |
| --- | --- | --- | --- | --- | --- | --- | --- | --- | --- | --- |
|  |  |  |  |  |  |  |  |  |  | (to be scored by researcher) |
| **Adulthood (17+ years)** | **Age** | **Frequency** | **Duration**  **(Years/Months)** | **Impact**  **(0-10)**  **Then Now** | | **Support**  **(+)**  **(0-3)** | **Support**  **(-)**  **(0-3)** | **Powerlessness**  **(0-10)**  **Then Now** | | **Anomalous Experience**  **(Pre/Post/ Both)** |
|  |  |  |  |  |  |  |  |  |  | (to be scored by researcher) |

**Scoring:**

I am now going to ask you some questions about some difficult experiences you may or may not have experienced at home during childhood (0-17 years) and adulthood

**PSYCHOLOGICAL ABUSE AT HOME**

| **2.** | **Were you ever tormented or treated cruelly by a member of household?**  **Did anyone try to frighten you?**  **Did anyone try to humiliate you? (e.g. belittle you in front of others, ridicule you)**  **Did you ever feel that these punishments at home were totally unnecessary?**  **If yes, refer to prompts** | **Yes/No** |
| --- | --- | --- |

**Notes:**

.......................................................................................................................................................................................................................................................................................................................................................................................... ....................................................................................................................................................................................................................................................................................................................................................................................................................................................................................................................................................................................... .................................................................................................................................................................................................................................................................................................................................................................................................................................................................................................................................................................................................................................................................................................................................................................................... .......................................................................................................................................................................................................................................................................................................................................................................................... .............................................................................................................................................................................................

**Scoring:**

| **Childhood (0-17 years)** | **Age** | **Frequency** | **Duration**  **(Years/**  **Months)** | **Relationship (1 – 9)** | **Impact**  **(0-10)**  **Then Now** | | **Support**  **(+)**  **(0-3)** | **Support**  **(-)**  **(0-3)** | **Powerlessness**  **(0-10)**  **Then Now** | | **Anomalous Experience (Pre-, Post, Both)** |
| --- | --- | --- | --- | --- | --- | --- | --- | --- | --- | --- | --- |
|  |  |  |  |  |  |  |  |  |  |  | (to be scored by researcher) |
| **Adulthood (17+ years)** | **Age** | **Frequency** | **Duration (Years**  **/Months)** | **Relationship (1 – 9)** | **Impact**  **(0-10)**  **Then Now** | | **Support**  **(+)**  **(0-3)** | **Support**  **(-)**  **(0-3)** | **Powerlessness**  **(0-10)**  **Then Now** | | **Anomalous Experience (Pre-, Post, Both)** |
|  |  |  |  |  |  |  |  |  |  |  | (to be scored by researcher) |

**PARENTAL NEGLECT**

| **3.** | **Were your material, social, educational or emotional needs ever not met by your parents (caregivers) when growing up?**  **(e.g. a lack of interest in friends, schoolwork, not being able to parent if upset, and not providing basic material needs)**  **If yes, refer to prompts** | **Yes/No** |
| --- | --- | --- |

**Notes:**

.......................................................................................................................................................................................................................................................................................................................................................................................... ....................................................................................................................................................................................................................................................................................................................................................................................................................................................................................................................................................................................... .................................................................................................................................................................................................................................................................................................................................................................................................................................................................................................................................................................................................................................................................................................................................................................................... .......................................................................................................................................................................................................................................................................................................................................................................................... .............................................................................................................................................................................................

**Scoring:**

| **Childhood (0-17 years)** | **Age** | **Frequency** | **Duration**  **(Years/**  **Months)** | **Relationship (1 – 9)** | **Impact**  **(0-10)**  **Then Now** | | **Support**  **(+)**  **(0-3)** | **Support**  **(-)**  **(0-3)** | **Powerlessness**  **(0-10)**  **Then Now** | | **Anomalous Experience (Pre-, Post, Both)** |
| --- | --- | --- | --- | --- | --- | --- | --- | --- | --- | --- | --- |
|  |  |  |  |  |  |  |  |  |  |  | (to be scored by researcher) |

**PHYSICAL ABUSE AT HOME**

| **4.** | **Were you ever slapped on a number of occasions, sufficient to cause harm?**  **Were you ever hit repeatedly with an implement (such as a belt or stick) or punched, kicked or burnt by someone in the household?**  **Did you ever feel that these punishments at home were totally unnecessary?** | **Yes/No** |
| --- | --- | --- |

**If yes, refer to prompts**

**Notes:**

.......................................................................................................................................................................................................................................................................................................................................................................................... ....................................................................................................................................................................................................................................................................................................................................................................................................................................................................................................................................................................................... .................................................................................................................................................................................................................................................................................................................................................................................................................................................................................................................................................................................................................................................................................................................................................................................... .......................................................................................................................................................................................................................................................................................................................................................................................... .............................................................................................................................................................................................

**Scoring:**

| **Childhood (0-17 years)** | **Age** | **Frequency** | **Duration**  **(Years/**  **Months)** | **Relationship (1 – 9)** | **Impact**  **(0-10)**  **Then Now** | | **Support**  **(+)**  **(0-3)** | **Support**  **(-)**  **(0-3)** | **Powerlessness**  **(0-10)**  **Then Now** | | **Anomalous Experience (Pre-, Post, Both)** |
| --- | --- | --- | --- | --- | --- | --- | --- | --- | --- | --- | --- |
|  |  |  |  |  |  |  |  |  |  |  | (to be scored by researcher) |
| **Adulthood (17+ years)** | **Age** | **Frequency** | **Duration (Years**  **/Months)** | **Relationship (1 – 9)** | **Impact**  **(0-10)**  **Then Now** | | **Support**  **(+)**  **(0-3)** | **Support**  **(-)**  **(0-3)** | **Powerlessness**  **(0-10)**  **Then Now** | | **Anomalous Experience (Pre-, Post, Both)** |
|  |  |  |  |  |  |  |  |  |  |  | (to be scored by researcher) |

**THREAT OR ACTUAL ASSAULT**

The next few questions are about whether you have ever been threatened or assaulted.

**Did you have any such experiences? Yes/No**

**If yes, ask Item 5**

| **5.** | **At any time in your life, has anyone (including family members or friends) *threatened* to attack you with a weapon (a gun, knife, or some other weapon) *or* without a weapon but with the intent to kill or seriously harm you?**  **If yes, refer to prompts** | **Yes/No** |
| --- | --- | --- |

**Notes:**

.......................................................................................................................................................................................................................................................................................................................................................................................... ....................................................................................................................................................................................................................................................................................................................................................................................................................................................................................................................................................................................... .................................................................................................................................................................................................................................................................................................................................................................................................................................................................................................................................................................................................................................................................................................................................................................................... .......................................................................................................................................................................................................................................................................................................................................................................................... .............................................................................................................................................................................................

**Scoring:**

| **Childhood (0-17 years)** | **Age** | **Frequency** | **Duration**  **(Years/**  **Months)** | **Relationship (1 – 9)** | **Impact**  **(0-10)**  **Then Now** | | **Support**  **(+)**  **(0-3)** | **Support**  **(-)**  **(0-3)** | **Powerlessness**  **(0-10)**  **Then Now** | | **Anomalous Experience (Pre-, Post, Both)** |
| --- | --- | --- | --- | --- | --- | --- | --- | --- | --- | --- | --- |
|  |  |  |  |  |  |  |  |  |  |  | (to be scored by researcher) |
| **Adulthood (17+ years)** | **Age** | **Frequency** | **Duration (Years**  **/Months)** | **Relationship (1 – 9)** | **Impact**  **(0-10)**  **Then Now** | | **Support**  **(+)**  **(0-3)** | **Support**  **(-)**  **(0-3)** | **Powerlessness**  **(0-10)**  **Then Now** | | **Anomalous Experience (Pre-, Post, Both)** |
|  |  |  |  |  |  |  |  |  |  |  | (to be scored by researcher) |

| **6.** | **At any time in your life, has anyone (including family members or friends) ever attacked you with a weapon (a gun, knife, or some other weapon) *or* without a weapon but with the intent to kill or seriously harm you, regardless of whether you ever reported it?**  **If yes, refer to prompts** | **Yes/No** |
| --- | --- | --- |

**Notes:**

.......................................................................................................................................................................................................................................................................................................................................................................................... ....................................................................................................................................................................................................................................................................................................................................................................................................................................................................................................................................................................................... .................................................................................................................................................................................................................................................................................................................................................................................................................................................................................................................................................................................................................................................................................................................................................................................... .......................................................................................................................................................................................................................................................................................................................................................................................... .............................................................................................................................................................................................

**Scoring:**

| **Childhood (0-17 years)** | **Age** | **Frequency** | **Duration**  **(Years/**  **Months)** | **Relationship (1 – 9)** | **Impact**  **(0-10)**  **Then Now** | | **Support**  **(+)**  **(0-3)** | **Support**  **(-)**  **(0-3)** | **Powerlessness**  **(0-10)**  **Then Now** | | **Anomalous Experience (Pre-, Post, Both)** |
| --- | --- | --- | --- | --- | --- | --- | --- | --- | --- | --- | --- |
|  |  |  |  |  |  |  |  |  |  |  | (to be scored by researcher) |
| **Adulthood (17+ years)** | **Age** | **Frequency** | **Duration (Years**  **/Months)** | **Relationship (1 – 9)** | **Impact**  **(0-10)**  **Then Now** | | **Support**  **(+)**  **(0-3)** | **Support**  **(-)**  **(0-3)** | **Powerlessness**  **(0-10)**  **Then Now** | | **Anomalous Experience (Pre-, Post, Both)** |
|  |  |  |  |  |  |  |  |  |  |  | (to be scored by researcher) |

**SEXUAL ABUSE**

I am now going to ask you some questions about unwanted sexual experiences during childhood (-0-17 years) and adulthood.

|  | **Did you ever have any such experiences?**  **If yes, ask Item 7** | **Yes/No** |
| --- | --- | --- |
| **7.** | **Did anyone force or persuade you to have sexual intercourse against your wishes?**  **If yes, refer to prompts** | **Yes/No** |

**Notes:**

.......................................................................................................................................................................................................................................................................................................................................................................................... ....................................................................................................................................................................................................................................................................................................................................................................................................................................................................................................................................................................................... .................................................................................................................................................................................................................................................................................................................................................................................................................................................................................................................................................................................................................................................................................................................................................................................... .......................................................................................................................................................................................................................................................................................................................................................................................... .............................................................................................................................................................................................

**Scoring:**

| **Childhood (0-17 years)** | **Age** | **Frequency** | **Duration**  **(Years/**  **Months)** | **Relationship (1 – 9)** | **Impact**  **(0-10)**  **Then Now** | | **Support**  **(+)**  **(0-3)** | **Support**  **(-)**  **(0-3)** | **Powerlessness**  **(0-10)**  **Then Now** | | **Anomalous Experience (Pre-, Post, Both)** |
| --- | --- | --- | --- | --- | --- | --- | --- | --- | --- | --- | --- |
|  |  |  |  |  |  |  |  |  |  |  | (to be scored by researcher) |
| **Adulthood (17+ years)** | **Age** | **Frequency** | **Duration (Years**  **/Months)** | **Relationship (1 – 9)** | **Impact**  **(0-10)**  **Then Now** | | **Support**  **(+)**  **(0-3)** | **Support**  **(-)**  **(0-3)** | **Powerlessness**  **(0-10)**  **Then Now** | | **Anomalous Experience (Pre-, Post, Both)** |
|  |  |  |  |  |  |  |  |  |  |  | (to be scored by researcher) |

| **8.** | **Can you think of any other upsetting sexual experiences with a related adult or someone in authority e.g. teacher?**  **If yes, refer to prompts** | **Yes/No** |
| --- | --- | --- |

**Notes:**

.......................................................................................................................................................................................................................................................................................................................................................................................... ....................................................................................................................................................................................................................................................................................................................................................................................................................................................................................................................................................................................... .................................................................................................................................................................................................................................................................................................................................................................................................................................................................................................................................................................................................................................................................................................................................................................................... .......................................................................................................................................................................................................................................................................................................................................................................................... .............................................................................................................................................................................................

**Scoring:**

| **Childhood (0-17 years)** | **Age** | **Frequency** | **Duration**  **(Years/**  **Months)** | **Relationship (1 – 9)** | **Impact**  **(0-10)**  **Then Now** | | **Support**  **(+)**  **(0-3)** | **Support**  **(-)**  **(0-3)** | **Powerlessness**  **(0-10)**  **Then Now** | | **Anomalous Experience (Pre-, Post, Both)** |
| --- | --- | --- | --- | --- | --- | --- | --- | --- | --- | --- | --- |
|  |  |  |  |  |  |  |  |  |  |  | (to be scored by researcher) |
| **Adulthood (17+ years)** | **Age** | **Frequency** | **Duration (Years**  **/Months)** | **Relationship (1 – 9)** | **Impact**  **(0-10)**  **Then Now** | | **Support**  **(+)**  **(0-3)** | **Support**  **(-)**  **(0-3)** | **Powerlessness**  **(0-10)**  **Then Now** | | **Anomalous Experience (Pre-, Post, Both)** |
|  |  |  |  |  |  |  |  |  |  |  | (to be scored by researcher) |

| **9.** | 1. **Has anyone ever used physical force or threat of force to make you have some type of unwanted sexual contact with them?**   **If yes, refer to prompts** | **Yes/No** |
| --- | --- | --- |

**Notes:**

.......................................................................................................................................................................................................................................................................................................................................................................................... ....................................................................................................................................................................................................................................................................................................................................................................................................................................................................................................................................................................................... .................................................................................................................................................................................................................................................................................................................................................................................................................................................................................................................................................................................................................................................................................................................................................................................... .......................................................................................................................................................................................................................................................................................................................................................................................... .............................................................................................................................................................................................

**Scoring:**

| **Childhood (0-17 years)** | **Age** | **Frequency** | **Duration**  **(Years/**  **Months)** | **Relationship (1 – 9)** | **Impact**  **(0-10)**  **Then Now** | | **Support**  **(+)**  **(0-3)** | **Support**  **(-)**  **(0-3)** | **Powerlessness**  **(0-10)**  **Then Now** | | **Anomalous Experience (Pre-, Post, Both)** |
| --- | --- | --- | --- | --- | --- | --- | --- | --- | --- | --- | --- |
|  |  |  |  |  |  |  |  |  |  |  | (to be scored by researcher) |
| **Adulthood (17+ years)** | **Age** | **Frequency** | **Duration (Years**  **/Months)** | **Relationship**  **(1 – 9)** | **Impact**  **(0-10)**  **Then Now** | | **Support**  **(+)**  **(0-3)** | **Support**  **(-)**  **(0-3)** | **Powerlessness**  **(0-10)**  **Then Now** | | **Anomalous Experience (Pre-, Post, Both)** |
|  |  |  |  |  |  |  |  |  |  |  | (to be scored by researcher) |

**SECTION 2: DISCRIMINATION**

The following questions are asking about the way other people have treated you, or your beliefs about the way other people have treated you.

*[note to researcher- only ask prompts if information is not spontaneously given]*

| **10.** | **Have you ever been unfairly treated at work (e.g. being fired, denied a promotion or not hired for a job)?**   1. **If yes, refer to prompts** | **Yes/No** |
| --- | --- | --- |

**Notes:**

.......................................................................................................................................................................................................................................................................................................................................................................................... ....................................................................................................................................................................................................................................................................................................................................................................................................................................................................................................................................................................................... .................................................................................................................................................................................................................................................................................................................................................................................................................................................................................................................................................................................................................................................................................................................................................................................... .......................................................................................................................................................................................................................................................................................................................................................................................... .............................................................................................................................................................................................

**Scoring:**

| **Childhood (0-17 years)** | **Age** | **Frequency** | **Duration**  **(Years/**  **Months)** | **Reason**  **(1 – 7)** | **Impact**  **(0-10)**  **Then Now** | | **Support**  **(+)**  **(0-3)** | **Support**  **(-)**  **(0-3)** | **Powerlessness**  **(0-10)**  **Then Now** | | **Anomalous Experience (Pre-, Post, Both)** |
| --- | --- | --- | --- | --- | --- | --- | --- | --- | --- | --- | --- |
|  |  |  |  |  |  |  |  |  |  |  | (to be scored by researcher) |
| **Adulthood (17+ years)** | **Age** | **Frequency** | **Duration (Years**  **/Months)** | **Reason**  **(1 – 7)** | **Impact**  **(0-10)**  **Then Now** | | **Support**  **(+)**  **(0-3)** | **Support**  **(-)**  **(0-3)** | **Powerlessness**  **(0-10)**  **Then Now** | | **Anomalous Experience (Pre-, Post, Both)** |
|  |  |  |  |  |  |  |  |  |  |  | (to be scored by researcher) |

| **11.** | **Have you ever been unfairly stopped, questioned, threatened by police?**   1. **If yes, refer to prompts** | **Yes/No** |
| --- | --- | --- |

**Notes:**

.......................................................................................................................................................................................................................................................................................................................................................................................... ....................................................................................................................................................................................................................................................................................................................................................................................................................................................................................................................................................................................... .................................................................................................................................................................................................................................................................................................................................................................................................................................................................................................................................................................................................................................................................................................................................................................................... .......................................................................................................................................................................................................................................................................................................................................................................................... .............................................................................................................................................................................................

**Scoring:**

| **Childhood (0-17 years)** | **Age** | **Frequency** | **Duration**  **(Years/**  **Months)** | **Reason**  **(1 – 7)** | **Impact**  **(0-10)**  **Then Now** | | **Support**  **(+)**  **(0-3)** | **Support**  **(-)**  **(0-3)** | **Powerlessness**  **(0-10)**  **Then Now** | | **Anomalous Experience (Pre-, Post, Both)** |
| --- | --- | --- | --- | --- | --- | --- | --- | --- | --- | --- | --- |
|  |  |  |  |  |  |  |  |  |  |  | (to be scored by researcher) |
| **Adulthood (17+ years)** | **Age** | **Frequency** | **Duration (Years**  **/Months)** | **Reason**  **(1 – 7)** | **Impact**  **(0-10)**  **Then Now** | | **Support**  **(+)**  **(0-3)** | **Support**  **(-)**  **(0-3)** | **Powerlessness**  **(0-10)**  **Then Now** | | **Anomalous Experience (Pre-, Post, Both)** |
|  |  |  |  |  |  |  |  |  |  |  | (to be scored by researcher) |

| **12.** | **Have you ever been unfairly treated by the court system?**   1. **If yes, refer to prompts** | **Yes/No** |
| --- | --- | --- |

**Notes:**

.......................................................................................................................................................................................................................................................................................................................................................................................... ....................................................................................................................................................................................................................................................................................................................................................................................................................................................................................................................................................................................... .................................................................................................................................................................................................................................................................................................................................................................................................................................................................................................................................................................................................................................................................................................................................................................................... .......................................................................................................................................................................................................................................................................................................................................................................................... .............................................................................................................................................................................................

**Scoring:**

| **Childhood (0-17 years)** | **Age** | **Frequency** | **Duration**  **(Years/**  **Months)** | **Reason**  **(1 – 7)** | **Impact**  **(0-10)**  **Then Now** | | **Support**  **(+)**  **(0-3)** | **Support**  **(-)**  **(0-3)** | **Powerlessness**  **(0-10)**  **Then Now** | | **Anomalous Experience (Pre-, Post, Both)** |
| --- | --- | --- | --- | --- | --- | --- | --- | --- | --- | --- | --- |
|  |  |  |  |  |  |  |  |  |  |  | (to be scored by researcher) |
| **Adulthood (17+ years)** | **Age** | **Frequency** | **Duration (Years**  **/Months)** | **Reason**  **(1 – 7)** | **Impact**  **(0-10)**  **Then Now** | | **Support**  **(+)**  **(0-3)** | **Support**  **(-)**  **(0-3)** | **Powerlessness**  **(0-10)**  **Then Now** | | **Anomalous Experience (Pre-, Post, Both)** |
|  |  |  |  |  |  |  |  |  |  |  | (to be scored by researcher) |

| **13.** | 1. **Have you ever been unfairly treated or discriminated against by your neighbours or family?** 2. **If yes, refer to prompts** | **Yes/No** |
| --- | --- | --- |

**Notes:**

.......................................................................................................................................................................................................................................................................................................................................................................................... ....................................................................................................................................................................................................................................................................................................................................................................................................................................................................................................................................................................................... .................................................................................................................................................................................................................................................................................................................................................................................................................................................................................................................................................................................................................................................................................................................................................................................... .......................................................................................................................................................................................................................................................................................................................................................................................... .............................................................................................................................................................................................

**Scoring:**

| **Childhood (0-17 years)** | **Age** | **Frequency** | **Duration**  **(Years/**  **Months)** | **Reason**  **(1 – 7)** | **Impact**  **(0-10)**  **Then Now** | | **Support**  **(+)**  **(0-3)** | **Support**  **(-)**  **(0-3)** | **Powerlessness**  **(0-10)**  **Then Now** | | **Anomalous Experience (Pre-, Post, Both)** |
| --- | --- | --- | --- | --- | --- | --- | --- | --- | --- | --- | --- |
|  |  |  |  |  |  |  |  |  |  |  | (to be scored by researcher) |
| **Adulthood (17+ years)** | **Age** | **Frequency** | **Duration (Years**  **/Months)** | **Reason**  **(1 – 7)** | **Impact**  **(0-10)**  **Then Now** | | **Support**  **(+)**  **(0-3)** | **Support**  **(-)**  **(0-3)** | **Powerlessness**  **(0-10)**  **Then Now** | | **Anomalous Experience (Pre-, Post, Both)** |
|  |  |  |  |  |  |  |  |  |  |  | (to be scored by researcher) |

| **14.** | **Have you ever been unfairly treated when getting medical care?**   1. **If yes, refer to prompts** | **Yes/No** |
| --- | --- | --- |

**Notes:**

.......................................................................................................................................................................................................................................................................................................................................................................................... ....................................................................................................................................................................................................................................................................................................................................................................................................................................................................................................................................................................................... .................................................................................................................................................................................................................................................................................................................................................................................................................................................................................................................................................................................................................................................................................................................................................................................... .......................................................................................................................................................................................................................................................................................................................................................................................... .............................................................................................................................................................................................

**Scoring:**

| **Childhood (0-17 years)** | **Age** | **Frequency** | **Duration**  **(Years/**  **Months)** | **Reason**  **(1 – 7)** | **Impact**  **(0-10)**  **Then Now** | | **Support**  **(+)**  **(0-3)** | **Support**  **(-)**  **(0-3)** | **Powerlessness**  **(0-10)**  **Then Now** | | **Anomalous Experience (Pre-, Post, Both)** |
| --- | --- | --- | --- | --- | --- | --- | --- | --- | --- | --- | --- |
|  |  |  |  |  |  |  |  |  |  |  | (to be scored by researcher) |
| **Adulthood (17+ years)** | **Age** | **Frequency** | **Duration (Years**  **/Months)** | **Reason**  **(1 – 7)** | **Impact**  **(0-10)**  **Then Now** | | **Support**  **(+)**  **(0-3)** | **Support**  **(-)**  **(0-3)** | **Powerlessness**  **(0-10)**  **Then Now** | | **Anomalous Experience (Pre-, Post, Both)** |
|  |  |  |  |  |  |  |  |  |  |  | (to be scored by researcher) |

**SCORING GUIDE:**

| **Frequency** | **0**  **1**  **2**  **3**  **4** | Never  Rarely (once or twice)  Occasionally (more than twice, less than monthly)  Frequently (monthly+)  Very frequently (weekly+) | |
| --- | --- | --- | --- |
| **Duration** | Length of time the experience lasted in years, months, days. | | |
| **Support** |  | |  |
| **Positive Support** | **0 = None** | | No support received |
|  | **1 = Some** | | Brief or minimal support was received that was  limited helpfulness |
|  | **2 = Moderate** | | Satisfactory emotional or practical support from one (or more) person but may not have been enough to help participant deal with the event or experience |
|  | **3 = High** | | Satisfactory emotional and practical support received. Subject able to confide, felt supported by one (or more) who helped participant deal with the event or experience |
| **Negative Support** | **0 = None** | | Positive or neutral response |
|  | **1 = Some** | | Confiding ignored or some disbelief expressed |
|  | **2 = Moderate** | | Participant is accused of lying about the event or experience or insinuation that was to blame |
|  | **3 = High** | | Clear statement that participant is to blame or deserved what happened |

**PARTICIPANT RESPONSE CARD**

**Impact:**

“How much did this event/experience affect you at the time?”

0 1 2 3 4 5 6 7 8 9 10

|------------|------------|------------|------------|------------|------------|------------|------------|------------|------------|

Not at all A little Somewhat Quite a lot Totally

“How much does this event/experience affect you now?”

0 1 2 3 4 5 6 7 8 9 10

|------------|------------|------------|------------|------------|------------|------------|------------|------------|------------|

Not at all A little Somewhat Quite a lot Totally

**Powerlessness:**

“Did you feel powerless at the time of this experience?”

0 1 2 3 4 5 6 7 8 9 10

|------------|------------|------------|------------|------------|------------|------------|------------|------------|------------|

Not at all A little Somewhat Quite a lot Totally

“How powerless does this event/experience make you feel now?”

0 1 2 3 4 5 6 7 8 9 10

|------------|------------|------------|------------|------------|------------|------------|------------|------------|------------|

Not at all A little Somewhat Quite a lot Totally

**Relationship:**

**1=Both Parents**

**2=Mother**

**3=Father**

**4=Sibling**

**5=Other Relative**

**6=Family Friend**

**7=Peer**

**8=Authority Figure**

**9=Other (please specify)**

**Reason:**

**1=Gender**

**2=Race, Ethnicity**

**3=Religion**

**4=Mental Health Problems**

**5=Sexuality**

**6=Age**

**7=Other (please specify)**

**Table S1**

*Total Scores Number of Victimisation Experiences*

|  | | Controls (n=83) | Non-clinical (n=92) | Clinical (n=82) | Test statistics |
| --- | --- | --- | --- | --- | --- |
| Interpersonal trauma | | | | | |
|  | Lifetime: mean (SD); median (IQR) | 3.52 (2.93);  3.00 (3.50) | 4.75 (3.83);  4.00 (4.00) | 3.87 (3.18);  3.50 (4.00)* | *H* (2)=6.38, *p*=0.04, *ε^2^*=0.025  Control vs. non-clinical: *z*=-2.41, *p_adj_=*0.047, *r*=0.151;  Control vs. clinical: *z*=-0.59, *p_adj_=*0.914, *r*=0.037;  Non-clinical vs. clinical: *z*=1.79, *p_adj_=*0.204, *r*=0.112 |
|  | Childhood: mean (SD); median (IQR)^a^ | 2.42 (2.23);  2.00 (3.00) | 3.02 (2.38);  2.50 (3.00) | 2.64 (2.52);  2.00 (3.00)† | *H* (2)=4.80, *p*=0.09, *ε^2^*=0.017  Control vs. non-clinical: *z*=-1.96, *p_adj_=*0.144, *r*=0.122;  Control vs. clinical: *z*=-0.35, *p_adj_=*0.979, *r*=0.022;  Non-clinical vs. clinical: *z*=1.58, *p_adj_=*0.304, *r*=0.099 |
|  | Adulthood: mean (SD); median (IQR) | 1.10 (1.38);  1.00 (2.00) | 1.73 (2.02);  1.00 (2.00) | 1.23 (1.29);  1.00 (2.00)* | *H* (2)=5.26 *p*=0.07, *ε^2^*=0.021  Control vs. non-clinical: *z*=-2.26, *p_adj_=*0.070, *r*=0.142;  Control vs. clinical: *z*=-0.82, *p_adj_=*0.799, *r*=0.051;  Non-clinical vs. clinical: *z*=1.40, *p_adj_=*0.409, *r*=0.088 |
| Perceived discrimination | | | | | |
|  | Lifetime: mean (SD); median (IQR)^a^ | 0.96 (1.23);  1.00 (2.00) | 1.18 (1.37);  1.00 (2.00) | 1.87 (1.68);  2.00 (3.00) | *H* (2)=16.15, *p*<0.001, *ε^2^*=0.063  Control vs. non-clinical: *z*=-1.04, *p_adj_=*0.653, *r*=0.065;  Control vs. clinical: *z*=-3.87, *p_adj_=*0.0003, *r*=0.241;  Non-clinical vs. clinical: *z*=-2.93, *p_adj_=*0.010, *r*=0.183 |
|  | Childhood: mean (SD); median (IQR) | 0.08 (0.32);  0.00 (0.00) | 0.12 (0.36);  0.00 (0.00) | 0.16 (0.46);  0.00 (0.00) | *H* (2)=1.66, *p*=0.44, *ε^2^*=0.006  Control vs. non-clinical: *z*=-0.77, *p_adj_=*0.825, *r*=0.048;  Control vs. clinical: *z*=-1.28, *p_adj_=*0.489, *r*=0.08081;  Non-clinical vs. clinical: *z*=-0.545, *p_adj_=*0.929, *r*=0.034 |
|  | Adulthood: mean (SD); median (IQR) | 0.88 (1.10);  0.00 (2.00) | 1.07 (1.29;  1.00 (2.00) | 1.71 (1.57);  2.00 (3.00) | *H* (2)=15.10, *p*<0.001, *ε^2^*=0.059  Control vs. non-clinical: *z*=-0.80, *p_adj_=*0.808, *r*=0.050;  Control vs. clinical: *z*=-3.68, *p_adj_=*0.0007, *r*=0.229;  Non-clinical vs. clinical: *z*=-2.97, *p_adj_=*0.009, *r*=0.185 |

*Note.* Sidak-adjusted p-values are reported for individual group comparisons. ES for overall group differences are reported as *ε ^2^* (0.01 small, 0.06 moderate, 0.14 large; [1]). ES for individual group comparisons were calculated as *r=*$z/\surd n$, where *z*=standardized test statistic, and *n*=number of observations (0.1 small, 0.3 moderate, 0.5 large; [2]).
* Two participants missing. † One participant missing.  ^a^ Scores on childhood interpersonal trauma and lifetime perceived discrimination previously reported [3].

**References**

[1] Kirk RE. Practical Significance: A Concept Whose Time Has Come. Educ Psychol Meas 1996;56:746–59. https://doi.org/10.1177/0013164496056005002.

[2] Cohen J. Statistical power analysis for the behavioral sciences. 1988.

[3] Peters E, Ward T, Jackson M, Morgan C, Charalambides M, McGuire P, et al. Clinical, socio-demographic and psychological characteristics in individuals with persistent psychotic experiences with and without a “need for care.” World Psychiatry 2016;15:41–52. https://doi.org/10.1002/WPS.20301.
